# Soft X-Ray Irradiation Effects of Li_2_O_2_, Li_2_CO_3_ and Li_2_O Revealed by Absorption Spectroscopy

**DOI:** 10.1371/journal.pone.0049182

**Published:** 2012-11-07

**Authors:** Ruimin Qiao, Yi-De Chuang, Shishen Yan, Wanli Yang

**Affiliations:** 1 School of Physics, Shandong University, Jinan, Shandong, China; 2 Advanced Light Source, Lawrence Berkeley National Laboratory, Berkeley, California, United States of America; Boston College, United States of America

## Abstract

Li_2_O_2_, Li_2_CO_3_, and Li_2_O are three critical compounds in lithium-air and lithium-ion energy storage systems. Extensive measurements have been carried out to study the chemical species and their evolutions at difference stages of the device operation. While x-ray spectroscopy has been demonstrated to be one of the most powerful tools for such purpose, no systematic study on the irradiation effects have been reported. Here we carry out extensive time, position, and irradiation dependent Li *K*-edge soft x-ray absorption spectroscopy on these compounds with so far the best energy resolution. The ultra-high resolution in the current study allows the features in the absorption spectra to be well-resolved. The spectral lineshape thus serves as the fingerprints of these compounds, enabling the tracking of their evolution under x-ray irradiation. We found that both Li_2_O_2_ and Li_2_CO_3_ evidently evolve towards Li_2_O under the soft x-ray irradiation with Li_2_CO_3_ exhibiting a surprisingly higher sensitivity to x-rays than Li_2_O_2_. On the other hand, Li_2_O remains the most stable compound despite experiencing substantial irradiation dose. We thus conclude that high resolution soft x-ray spectroscopy could unambiguously fingerprint different chemical species, but special cautions on irradiation effects would be needed in performing the experiments and interpreting the data properly.

## Introduction

At the heart of modern sustainable energy applications are the high performance energy storage systems. The demand of revolutionizing the current energy carriers, e.g. fossil fuel, has become ever more pressing especially in the utilization of intermittent renewable energy sources [1] and the realization of electric vehicles (EVs) [2]. Among all current electrochemical energy storage systems, Li-ion batteries have been extensively used in the portable electronic markets ever since their first commercialization by Sony in 1991 [3], and they are expected to remain the dominance in the near future if the safety and performance can be improved to match the requirements of Evs [4]–[6]. In the meantime, scientific attention has been gradually shifted to the next generation energy storage media beyond Li-ion, which could potentially provide much higher specific energy and energy density. One such example is the Li-air battery systems with theoretically several times higher specific energy than that of the Li-ion systems [7]–[14].

However, for both Li-ion and Li-air electrochemical systems, the critical understandings on the fundamental mechanism of battery operations remain incomplete and this severely hinders the rational and speedy improvements on the performance of these energy storage devices. Li-ion battery operates under non-equilibrium states far above the thermodynamic stability of electrolyte, or sometimes even electrodes [6]. In reality, a battery always operates in an alternative way by forming a passivating layer on the surface of the electrodes. This passivating layer, typically around 20–50 nm thick and is known as the solid-electrolyte interphase (SEI), protects electrolyte from further reduction/oxidation. Almost all Li-ion batteries on the market rely on such SEI mechanism for retaining their stability. For example, in the commercially available LiCoO_2_/Graphite batteries, the graphite based anode turns to reduce the electrolyte, leading to serious stability issues that have frustrated the practical application for decades until the proper electrolyte compounds were found [15]. It is now known that one component of the electrolyte, ethylene carbonate, decomposes and forms a passivating SEI layer on the surface of the carbon anode. This SEI layer, although consumes electrolyte for its formation, does provide the kinetic stability for protecting the electrolyte from further decomposition. SEI often contains Li_2_O, Li_2_O_2_, Li_2_CO_3_, fluorides and other organic compounds. However, the dual-functionality and controlling [16], [17] of SEI in batteries remain elusive and a hot topic in Li-ion battery researches [4], [18]–[24].

The principle of Li-air batteries is the catalytic reactions between lithium and oxygen, which is distinct from the insertion/extraction mechanism in the Li-ion batteries. The reversible oxidation of lithium at the anode and reduction of oxygen at the cathode could potentially yield an ultra-high energy capacity. However, the battery module suffers poor cycling performance, high-rate output, as well as stability issues [14], which require extensive further studies on the chemical nature during the battery operations. Besides the much discussed Li_2_O_2_ and Li_2_O as the critical species in the lithium-oxygen reaction [8], [11], [25]–[27], very recent reports [28]–[31] have suggested the presence of Li_2_CO_3_ in the discharge products if carbonate based electrolytes are used. At this time, the details of the lithium-oxygen reactions involved in Li-air batteries remains unclear.

Because of the importance of Li_2_O, Li_2_O_2_, and Li_2_CO_3_ in both Li-ion SEI and Li-air battery systems, there have been extensive efforts in studying the chemical compositions and the phase evolution of these compounds. In particular, electron energy loss spectroscopy (EELS) [32], hard X-ray non-resonant inelastic X-ray scattering (NIXS) [33]–[35] and soft X-ray absorption spectroscopy (XAS) [25], [36], [37] are used to probe the unoccupied electronic states that are sensitive to the local environment of lithium ions. Among these techniques, soft X-ray XAS typically offers the best experimental resolution with its inherent elemental, chemical and bonding sensitivity by tuning the x-rays from synchrotron sources across the absorption edges. Indeed, high-resolution soft x-ray absorption studies have captured a lot of research interests in fingerprinting these lithium compounds [25], [37]. On the other hand, due to the intrinsic nature of soft x-rays on both penetration depth and high cross-section of interacting with electrons, it often encounters more serious irradiation damage effects than hard x-rays. Specifically, the secondary electrons generated from soft x-ray photon-excitation process could strongly interact with the molecular bonding on surface, leading to undesired species. Such effects have been broadly observed in the organic molecular systems in XAS [38], [39], as well as X-ray photoelectron spectroscopy (XPS) [40]. Although the Li compounds studied in this work are considered ionic systems that are typically stable under x-rays, it is important to address the possible soft x-ray irradiation effects in these compounds in order to obtain accurate XAS spectra for understanding the nature of the battery systems. Not much attention has been paid to this essential topic, and an experimental report is still missing.

In this work, we present a systematic study with position, time and irradiation dependent Li *K*-edge soft x-ray XAS spectroscopy on Li_2_O, Li_2_O_2_, and Li_2_CO_3_ compounds. The experiments were done at beamline 4.0.3 (the milli-eV energy resolution beamline, MERLIN) of the Advanced Light Source (ALS), Lawrence Berkeley National Laboratory (LBNL). The ultra-high resolution enables the features in the XAS spectra to be well-resolved. More importantly, the spectra of both Li_2_O_2_ and Li_2_CO_3_ evolve unambiguously towards Li_2_O under soft x-ray irradiation, with Li_2_CO_3_ exhibiting a surprisingly higher sensitivity to x-rays than Li_2_O_2_. This work provides the clear experimental evidences of decomposition of Li_2_O_2_ and Li_2_CO_3_ into the final product of Li_2_O through soft x-ray irradiation.

## Experimental

The Li *K*-edge XAS measurements were conducted at the elliptically polarizing undulator (EPU) beamline 4.0.3 (MERLIN) [41] of the Advanced Light Source (ALS), Lawrence Berkeley National Laboratory (LBNL). The MERLIN beamline was newly constructed with a 1.9 m long, 90 mm period quasi-periodic EPU and spherical grating monochromator. It delivers the photon beam with energies ranging from 10 eV to ∼150 eV onto the sample and with better than 0.01 eV (10 meV) energy resolution, the photon flux is about 10^11^ photons per second. The complimentary O *K*-edge XAS spectra were measured at the undulator beamline 8.0.1 [42], where the intense photon beam from a spherical grating monochromator gives an energy resolution better than 0.2 eV at 500–550 eV. We note that, for the Li *K*-edge XAS, the ultra-high energy resolution offers a unique opportunity to experimentally determine the core-hole life time, because most of the broadening of spectral features is from the thermal effect and the intrinsic core-hole lifetime.

Chemicals in powder form with highest possible purity were purchased from Sigma-Aldrich. The powder samples were pressed into pellets (for Li *K*-edge measurement) or onto a conducting carbon tape (for O *K*-edge measurement) in the N_2_ glove box before loaded into the ultra-high vacuum chamber with base pressure better than 5×10^−10^ Torr. XAS experiments were performed at room temperature and spectra were recorded in bulk-sensitive total fluorescence yield mode (TFY) using a photodiode detector. All the data shown were normalized to the photon flux measured by the photocurrent of an upstream gold mesh. The probing depth is around tens of nanometers for Li-*K* and on the order of hundreds of nanometers for O-*K*.

## Results and Discussion

**Figure 1:**
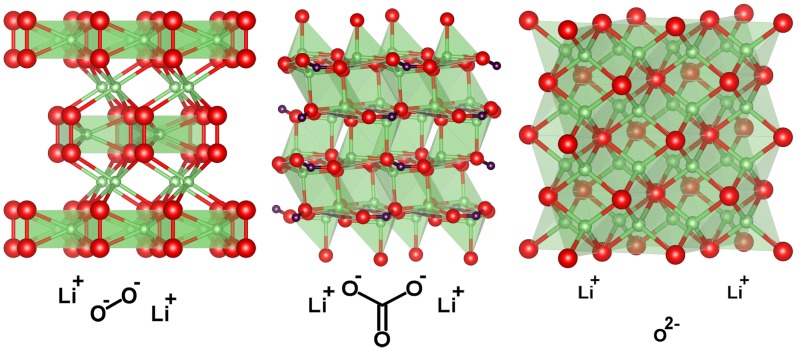
Crystal structure of Li_2_O_2_, Li_2_CO_3_, and Li_2_O. Red (largest) spheres represent oxygen atoms, green (smaller) spheres represent lithium atoms and purple (smallest) spheres are carbon atoms. Li-O polygons are drawn for better view.

As shown in figure 1, Li_2_O_2_ [43], [44] crystallizes in a P63/mmc hexagonal space group where the peroxide anions are arranged in an alternating ABAB stacking. There are two distinct Li^+^ sites: one is in the same layer as the peroxide anions and the other one is in between the peroxide layers. The crystal structure of Li_2_CO_3_ [45] (monoclinic, space group C2/c) is composed of nearly planar CO_3_^2−^ anions and Li^+^ cations tetrahedrally coordinated to oxygen atoms. This crystal structure consists of staggered Li_2_CO_3_ units. Li_2_O adopts a cubic crystal structure (space group Fm-3m), wherein lithium is coordinated to four oxygen anions and each O^2−^ ion is surrounded by eight Li^+^ ions. These three inorganic compounds are ionic insulators with strong Li-O bonds, and naively, one would not expect to observe pronounced x-ray irradiation damage.

**Figure 2:**
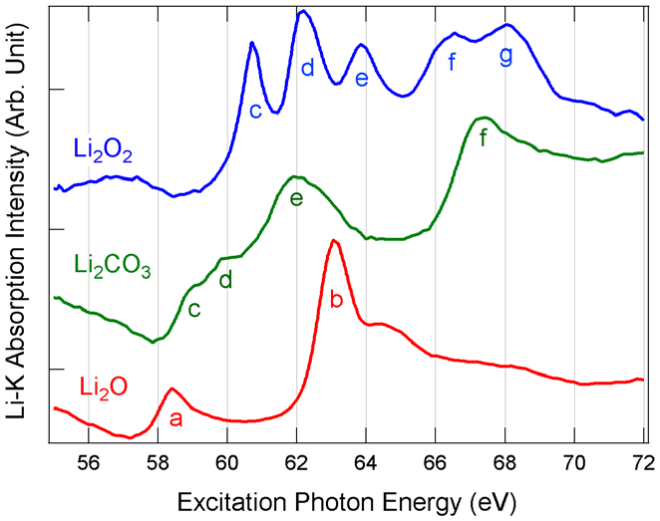
Li *K*-edge XAS spectra of Li_2_O_2_, Li_2_CO_3_, and Li_2_O.

Li *K*-edge XAS spectra collected from the fresh spots on three lithium compounds, Li_2_O_2_, Li_2_CO_3_, and Li_2_O, are shown in Figure 2. First of all, the distinctively different absorption features clearly demonstrate that Li *K*-edge XAS can be used to unambiguously fingerprint those chemical species in lithium batteries, as previously reported [25], [37]. Secondly, it is important to note that the peak width is not limited by the beamline instrumental resolution, but mostly by the lithium *1s* core-hole lifetime and thermal broadening [36], [46]. Since the Li *1s* core-hole is not well screened and has strong interaction with the excited *2p* electron, the excitonic effect can play an important role in the XAS spectra. In general, the excitonic effect would create sharp absorption features at lower energy and reduce the intensity at higher energy [47], as especially shown by the sharp features denoted a, b (red) in Li_2_O and c-g (blue) in Li_2_O_2_ in Fig. 2 [34], [46].

**Figure 3:**
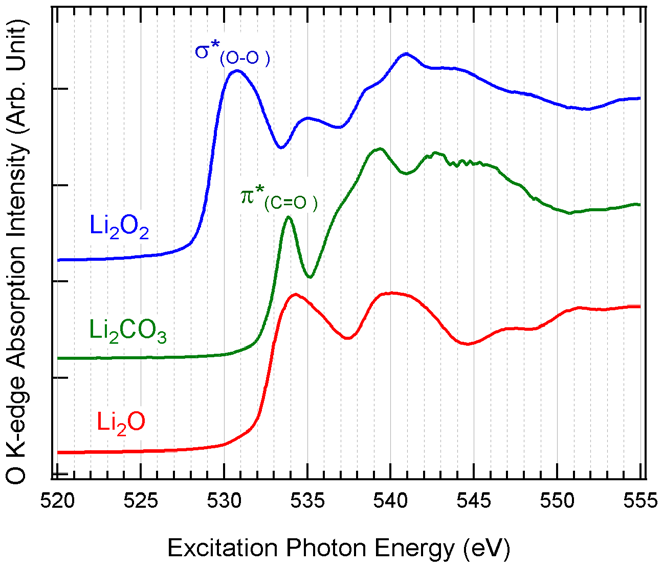
O *K*-edge XAS spectra of Li_2_O_2_, Li_2_CO_3_, and Li_2_O.

Figure 3 shows the O *K*-edge XAS spectra of Li_2_O_2_, Li_2_CO_3_ and Li_2_O. One significant difference between O and Li *K*-edge XAS spectra is that the O *1s* core hole is now well-screened compared with the shallow Li *1s* core hole. Thus O-*K* spectra are less affected by excitonic effect and better reflect the partial density of states of unoccupied O *2p* orbitals. All three lithium compounds display rather different absorption spectra due to variations in the local oxygen environment. As discussed previously, the first absorption feature on the XAS spectrum of Li_2_O_2_ is from electron transition to the orbital with largely σ* (O-O) character [48], while the sharp absorption peak of Li_2_CO_3_ can be assigned to be transition to π* (C = O) orbital [49]. It is worthy to note that the leading edge of Li_2_O_2_ XAS spectrum is about 3.5eV lower than that of Li_2_CO_3_ and Li_2_O, suggesting that the conduction band minimum of Li_2_O_2_ is lower than that of Li_2_CO_3_ and Li_2_O due to its O-O bond. Therefore, the O *K*-edge XAS spectra can also be used to fingerprint the chemical species and be complimentary to the Li *K*-edge XAS [48], [49].

**Figure 4:**
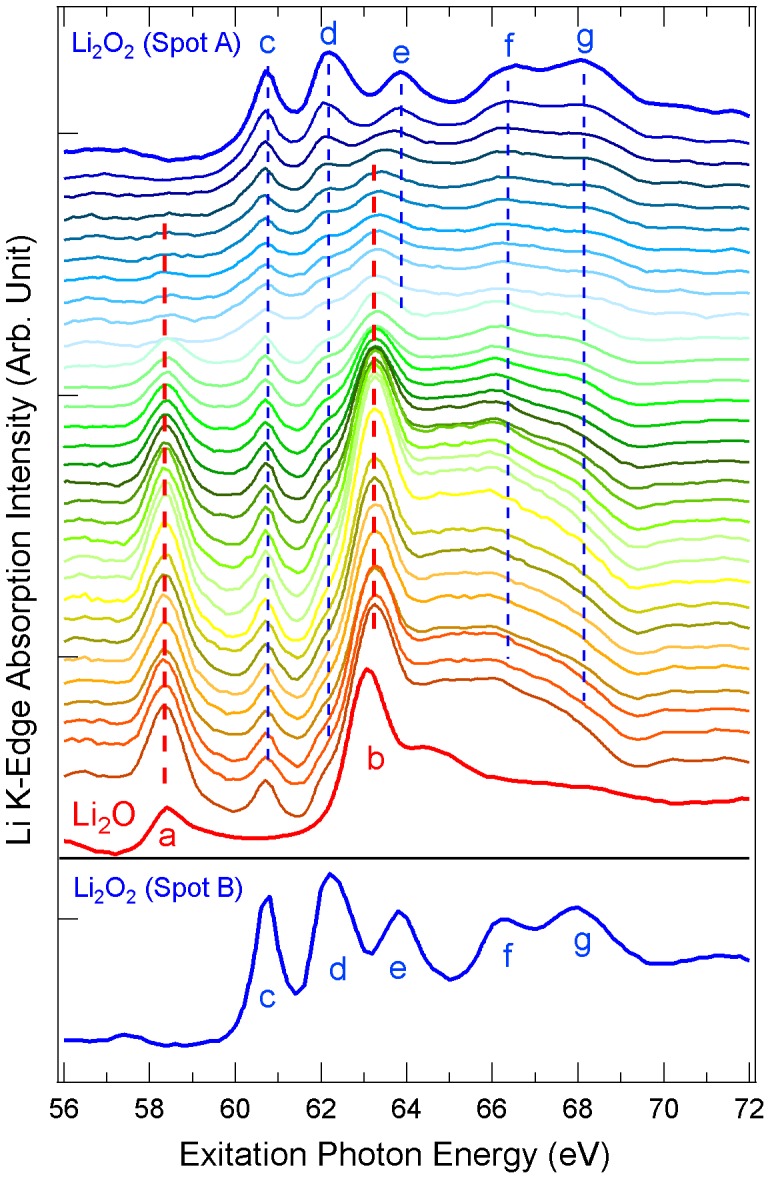
Soft x-ray irradiation effect on Li_2_O_2_ revealed by Li *K*-edge XAS spectra. (top) The XAS spectrum of Li_2_O_2_ evolves towards that of the Li_2_O upon increasing the radiation exposure. The top spectrum is the first one collected from spot A. From top to bottom, the first ten spectra were collected every ten minutes, with an hour of x-ray exposure at the same spot to maximize the dosage, then the measurements resumed with again ten minute each spectrum. The bottom red spectrum is collected on Li_2_O for comparison. (bottom) Li_2_O_2_ Li *K*-edge XAS spectrum measured from a new spot B on the same sample stored in the ultra-high vacuum chamber for one week.

**Figure 5:**
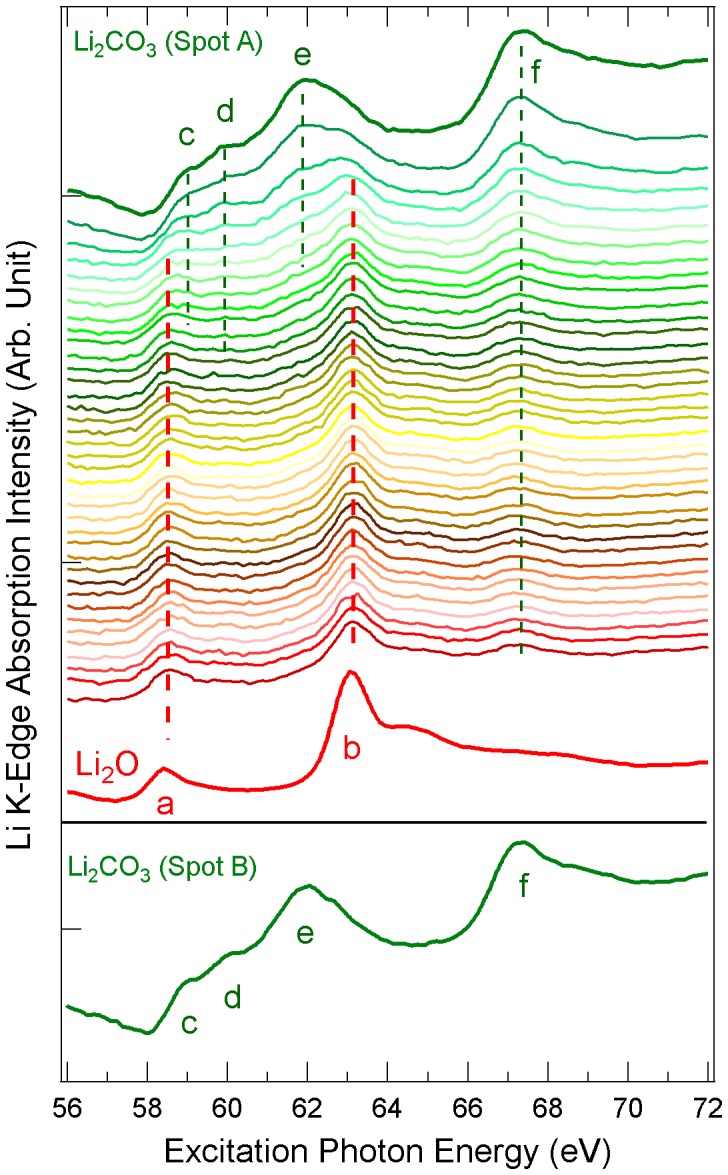
Soft x-ray irradiation effect on Li_2_CO_3_ revealed by Li *K*-edge XAS spectra. The XAS lineshape of Li_2_CO_3_ (from top to bottom) evolve towards that of Li_2_O (red) after exposed to the soft x-rays. Each spectrum was taken with 10 minute x-ray exposure. The bottom panel shows the Li_2_CO_3_ Li *K*-edge XAS spectrum collected from a new spot B on the same sample.

The central results of this work on irradiation effects of Li_2_O_2_ and Li_2_CO_3_ are presented in Fig. 4 and Fig. 5, respectively. Figure 4 shows the irradiation dependent Li *K*-edge XAS spectra of Li_2_O_2_, in comparison with Li_2_O. The Li_2_O_2_ XAS spectra (from top to bottom) were recorded from spot A on the sample by repeating the photon energy scan every ten minutes. In order to maximize the irradiation effect, after the 10^th^ spectrum, the sample was left exposed to x-rays for one hour, and then the measurement resumed. It is evident that the XAS spectrum of Li_2_O_2_ evolves with respect to the X-ray radiation exposure. All the absorption features of the pristine Li_2_O_2_ sample, marked by blue dashed lines (c-g), become weaker with irradiation, and most of them are diminished except for feature c. In the meantime, new absorption peaks labeled a and b (red lines) are enhanced with increased irradiation dose. These enhanced features are in good agreement with the ones in Li_2_O (Fig. 2). Therefore, it is evident that Li_2_O_2_ gradually decomposes to Li_2_O under soft x-ray irradiation.

To make sure that the change of spectral lineshape is from the exposure to soft x-rays instead of surface degradation in ultra-high vacuum, we took the XAS spectrum from a different spot B on the same Li_2_O_2_ sample that had been stored in the vacuum chamber for one week. As shown in figure 4 (bottom), the spectrum resembles the top one measured on the fresh surface. The similarity indicates that the sample surface is stable in high-vacuum, and the observed decomposition of Li_2_O_2_ is indeed induced by the X-ray irradiation.

Figure 5 shows the Li *K*-edge XAS spectra of Li_2_CO_3_ collected under the same condition and time scale as that for Li_2_O_2_. To our surprise, the XAS of Li_2_CO_3_ changes quickly with exposure to soft x-rays. The features from Li_2_CO_3_ (green c-f) quickly fade out in 30 minutes and the Li_2_O features (red a and b) can already be seen in the second spectrum (less than 20 minutes of exposure). The Li_2_O features dominate the overall lineshape from the fourth spectrum. We would like to point out that 10 to 60 minutes of exposure to soft x-rays with a photon flux on the order of 10^10^–10^11^ photons per second is typical for soft x-ray experiments, during which, the Li_2_CO_3_ may be decomposed and produce Li_2_O as suggested by the data in Figure 5.

**Figure 6:**
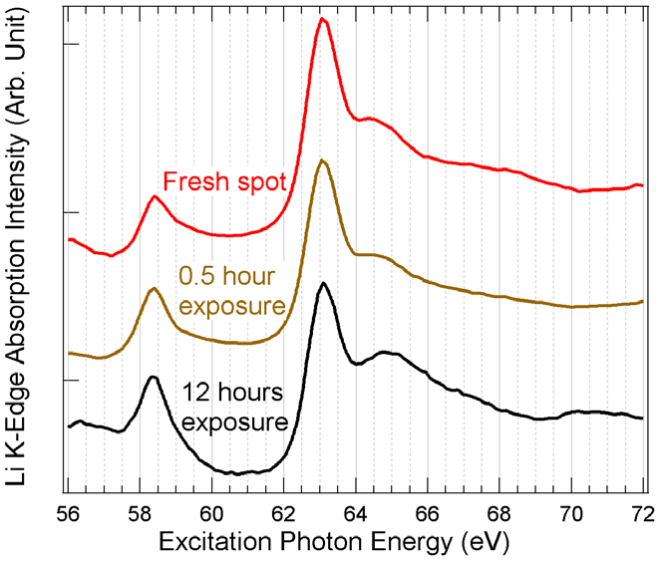
Li *K*-edge XAS spectra of Li_2_O. The spectra were collected on fresh surface, the same spot with 0.5 hour soft x-ray exposure, and 12 hours of soft x-ray exposure.

Finally, we confirm that Li_2_O is the only stable phase under soft x-ray irradiation. Figure 6 shows the Li *K*-edge XAS of Li_2_O over the course of 12-hour x-ray exposure, and yet the spectra remain nearly identical. Based on our experimental results, the irradiation induced decomposition of Li_2_O_2_ and Li_2_CO_3_ can be expressed as 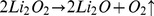 and 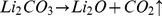.

Although the radiation damage effects are broadly investigated in organic systems, such as biomolecules and polymers [39], [50], [51], inorganic compounds are often considered resistant to soft x-ray irradiation damage [38]. For the irradiation effect of Li_2_O_2_ and Li_2_CO_3_ reported in this work, the decomposition may stem from several factors. Overall, both systems are ionic compounds dominated by insulating nature. The lack of enough conduction electrons prevents the quick restoration from irradiated states through electrical neutrality. In addition to the many scenarios based on x-ray excitations of core-hole, secondary electrons, electron-hole pair, and broken surface bonds, it is also well known that some energy losses from x-ray photons to phonons lead to local heat. Interestingly, the X-ray induced decomposition reaction has the same product as the heat induced reaction, in which both Li_2_O_2_ and Li_2_CO_3_ would decomposed to Li_2_O at temperature range of 200–450°C and 730–1270°C, respectively. Actually, Li_2_O_2_ is a strong oxidant that could be reduced to Li_2_O under various circumstances, but our results show that Li_2_O_2_ is only slowly reduced to Li_2_O under soft x-rays. On the other hand, Li_2_CO_3_ is more stable under ambient condition, yet it exhibits a higher degree of susceptibility to x-ray irradiation.

### Conclusions

In summary, we have performed a detailed study on the irradiation effects of Li_2_O_2_, Li_2_CO_3_ and Li_2_O. High resolution XAS spectra, capable of revealing distinct spectral features associated with different chemical species, allow us to track the chemical evolution from x-ray irradiation effects. We found both Li_2_O_2_ and Li_2_CO_3_ show clear evidence of decomposition with soft x-ray exposure with Li_2_CO_3_ exhibiting a surprisingly higher degree of sensitivity than the Li_2_O_2_. For both systems, the final product of decomposition is Li_2_O, which is rather stable against soft x-ray irradiation. The data presented in this work demonstrates the potential of soft x-ray XAS for studying the chemical nature and reaction pathway of the lithium compounds; however, it also suggests that experiments and data analysis should be performed with careful considerations of irradiation effects.
